# Diagnostic, Prognostic, and Predictive Biomarkers in Breast Cancer

**DOI:** 10.1155/2020/1835691

**Published:** 2020-03-07

**Authors:** Chia-Jung Li, Hui-Ming Chen, Ji-Ching Lai

**Affiliations:** ^1^Department of Obstetrics and Gynecology, Kaohsiung Veterans General Hospital, Kaohsiung, Taiwan; ^2^Institute of BioPharmaceutical Sciences, National Sun Yat-Sen University, Kaohsiung, Taiwan; ^3^Houston Methodist Research Institute, Houston, TX, USA; ^4^Department of Medical Research and Development, Chang Bing Show Chwan Memorial Hospital, Changhua, Taiwan

Breast cancer is the most frequently diagnosed cancer type and the second leading cause of cancer-related deaths among women worldwide. The causes of breast cancer are not yet fully known, although a number of risk factors have been identified. Tumor biomarker is a term used to describe potential markers of cancer development and progression. As we explore these biomarkers further, we must try to understand the underlying mechanisms of tumor development, as we move along the path to discovery of novel therapies that will increase our ability to offer personalized patient care in the future. With the migration of advanced high throughput technologies, such as Next Generation Sequencing from clinical practice, biomarker research and discovery are poised to explode once again. Translation of novel biomarkers into clinical practice and diagnostic laboratories is coupled with regulatory and administrative requirements that must be met, while collaboration between research institutions, industry, and the private sector drives further advancements in the field of breast cancer biomarker discovery and application.

The aim of this special issue is to provide new findings regarding molecular pathways and biomarkers that could improve the diagnosis and the prognostic classification of breast cancers, their application in the clinical setting, and their potential utility in personalized patient therapy. The total of submissions is 50. After single-blind peer review by at least two reviewers, 14 papers were finally accepted to be published. The accepted rate is 28%. The average number of authors for each accepted paper is 7. The affiliated institutes of the authors are from Brazil, China, Italy, Iraq, Jordan, Portugal, Spain, Saudi Arabia, Taiwan, the UK, and the USA. These accepted papers can be organized in different groups. The focus of the first group of articles is on prognosis and therapy in breast cancer. The findings of the paper titled “Exploring the Role of Breast Density on Cancer Prognosis among Women Attending Population-Based Screening Programmes” by Domingo et al. reveal that increased breast density was associated with worse survival outcomes among women participating in breast cancer screening. The paper titled “Attenuated Total Reflection-Fourier Transform Infrared (ATR-FTIR) Spectroscopy Analysis of Saliva for Breast Cancer Diagnosis” by Ferreira et al. showed ATR-FTIR spectroscopy can be used in saliva samples to discriminate breast cancer patients from benign patients and healthy subjects. The paper titled “Dovitinib Triggers Apoptosis and Autophagic Cell Death by Targeting SHP-1/p-STAT3 Signaling in Human Breast Cancers” by Chiu et al. has provided the evidence for anticancer effect of dovitinib to suggest it as a potential target for breast cancer therapy. The focus of the second group of articles is on biomarkers in breast cancer. The paper titled “A Novel Role for Cathepsin S as a Potential Biomarker in Triple Negative Breast Cancer” by Wilkinson et al. investigated the expression profile of Cathepsin S in breast cancer patients. The paper titled “Identification of Cell-Free Circulating MicroRNAs for the Detection of Early Breast Cancer and Molecular Subtyping” by Souza et al. identified the molecular signature miRNA as noninvasive biomarkers in patients with breast cancer. The paper titled “CD133 in Breast Cancer Cells: More Than a Stem Cell Marker” by Brugnoli et al. reviewed the value of CD133 as prognostic factor of malignant progression of breast cancer. The other four papers titled “*N*-Acetyltransferase 1 Knockout Elevates Acetyl Coenzyme A Levels and Reduces Anchorage-Independent Growth in Human Breast Cancer Cell Lines” by Stepp et al., “Overexpression of Kynurenine 3-Monooxygenase Correlates with Cancer Malignancy and Predicts Poor Prognosis in Canine Mammary Gland Tumors” by Chiu et al., “Notch Signaling Activation as a Hallmark for Triple Negative Breast Cancer Subtype” by Giuli et al., and “Human Mitotic Centromere-Associated Kinesin is Targeted by MicroRNA 485-5p/181c and Prognosticates Poor Survivability of Breast Cancer” by Lu et al. contributed different biomarkers of breast cancer which may help to assess prognosis or predictive indicators. The focus of the third group of articles is on genetic mutations in breast cancer. The paper titled “Association of ESR1 Mutations and Visceral Metastasis in Patients with Estrogen Receptor-Positive Advanced Breast Cancer from Brazil” by Reinert et al. observed an association of ESR1 mutations with metastasis. The paper titled “Role of Four ABC Transporter Genes in Pharmacogenetic Susceptibility to Breast Cancer in Jordanian Patients” by AL-Eitan et al. proposed the ABCB1 mutation associated with breast cancer in Jordanian Arabs. The paper titled “A Review of the Hereditary Component of Triple Negative Breast Cancer: High- and Moderate-Penetrance Breast Cancer Genes, Low-Penetrance Loci and the Role of Nontraditional Genetic Elements” by Ellsworth et al. described genes and genetic elements, which have been associated with increased risk of triple negative breast cancer. The paper titled “Pregnancy Hypertension and a Commonly Inherited IGF1R Variant (rs2016347) Reduce Breast Cancer Risk by Enhancing Mammary Gland Involution” by Powell et al. observed that statistically significant decrease in terminal duct lobular unit counts signifies increased breast epithelial involution in women with prior hypertension who inherited the TT genotype of IGF1R SNP (rs2016347).

In summary, the research papers cover a wide range of applications including potential diagnostics, predictions, and treatment. Furthermore, this special issue also includes regional genetic mutation, breast density on cancer prognosis, small RNA as a biomarker, surface marker of cancer stem cells, potential marker of cancer metastasis, and spectroscopy predicting the rate of cancer. This may be helpful in assessing prognostic or predictive indicators, as well as developing new therapies and new insights aimed at improving breast cancer.

*Chia-Jung Li* *Hui-Ming Chen* *Ji-Ching Lai*

